# Supplementary material for: Transcription-coupled repair of DNA–protein cross-links depends on CSA and CSB
Source: Nat Cell Biol. 2024 Apr 10;26(5):797–810. doi: 10.1038/s41556-024-01391-1 (PMC11098753; doi:10.1038/s41556-024-01391-1)
Supplement: Supplementary file 1 — Reporting Summary [file 41556_2024_1391_MOESM1_ESM.pdf]

nature portfolio

Corresponding author(s): Christopher J. Carnie, Stephen P. Jackson, Julian Stingele

Last updated by author(s): Feb 10, 2024

Reporting Summary

Nature Portfolio wishes to improve the reproducibility of the work that we publish. This form provides structure for consistency and transparency in reporting. For further information on Nature Portfolio policies, see our Editorial Policies and the Editorial Policy Checklist.

Statistics

For all statistical analyses, confirm that the following items are present in the figure legend, table legend, main text, or Methods section.

n/a	Confirmed	
☐	☒	The exact sample size (*n*) for each experimental group/condition, given as a discrete number and unit of measurement
☐	☒	A statement on whether measurements were taken from distinct samples or whether the same sample was measured repeatedly
☐	☒	The statistical test(s) used AND whether they are one- or two-sided<br>*Only common tests should be described solely by name; describe more complex techniques in the Methods section.*
☐	☒	A description of all covariates tested
☐	☒	A description of any assumptions or corrections, such as tests of normality and adjustment for multiple comparisons
☐	☒	A full description of the statistical parameters including central tendency (e.g. means) or other basic estimates (e.g. regression coefficient) AND variation (e.g. standard deviation) or associated estimates of uncertainty (e.g. confidence intervals)
☐	☒	For null hypothesis testing, the test statistic (e.g. *F*, *t*, *r*) with confidence intervals, effect sizes, degrees of freedom and *P* value noted<br>*Give P values as exact values whenever suitable.*
☒	☐	For Bayesian analysis, information on the choice of priors and Markov chain Monte Carlo settings
☒	☐	For hierarchical and complex designs, identification of the appropriate level for tests and full reporting of outcomes
☒	☐	Estimates of effect sizes (e.g. Cohen's *d*, Pearson's *r*), indicating how they were calculated

Our web collection on statistics for biologists contains articles on many of the points above.

Software and code

Policy information about availability of computer code

Data collection	Descriptions of the software used to collect data relating to this study are included throughout the Methods section.
Data analysis	Descriptions of the code used to analyse data as part of this study are included throughout the Methods section. For data presentation and analysis purposes, the following software packages were used: Harmony 5.2, ImageJ2 2.9.0/1.53t, CellProfiler 4.2.5, FastQC 0.11.8 and 0.11.9, TrimGalore 0.6.5, STAR 2.7.7a/gcc-8.3.1, HOMER tools 4.8.2, IGV 2.4.3, DIA-NN 1.8.2 beta 22, R 4.2.2 and 4.1.2, preprocessCore 1.60.0, MSnbase 2.24.0 MinDet method, limma, fastp 0.23.2, Bowtie2 2.4.5, Samtool 1.16.1, Deeptools 3.5.0 and 3.5.1, bedtools 2.30.0, bamCoverage, ggplot2 3.4.0, demux Illumina 3.0.9, MultiQC 1.11, cutadapt, Picard 2.20.3. As stated in the Code Availability statement, all analytical code for both upstream processing and downstream analysis and plot generation of DPC-seq data are publicly available at https://github.com/aldob/DPC-Seq.

For manuscripts utilizing custom algorithms or software that are central to the research but not yet described in published literature, software must be made available to editors and reviewers. We strongly encourage code deposition in a community repository (e.g. GitHub). See the Nature Portfolio guidelines for submitting code & software for further information.

Data

Policy information about availability of data

All manuscripts must include a data availability statement. This statement should provide the following information, where applicable:

- Accession codes, unique identifiers, or web links for publicly available datasets
- A description of any restrictions on data availability
- For clinical datasets or third party data, please ensure that the statement adheres to our policy

All raw and processed data relating to the CRISPRi screens, TTchem-seq, DPC-seq and CUT&Tag experiments described in Figures 1, 4, 6 and 7 and Extended Data Figures 8 and 9 have been uploaded to ArrayExpress under accession number E-MTAB-12912. As described, DPC-seq data were compared to publicly available RPB1 ChIP-seq and ATAC-seq data accessible through the GEO with the accession numbers GSE14179861 and GSE209659, respectively. The UCSC genome database was used to access Human Genome 38 for read alignments in TTchem-seq. The mass spectrometry proteomics data have been deposited to the ProteomeXchange Consortium via the PRIDE85 partner repository with the dataset identifier PXD047668. DrugZ analysis outputs from CRISPRi screens shown in Fig. 1 and Extended Data Fig. 1 is provided in Supplementary Tables 1-2. Source data has been provided in Source Data. All other raw data supporting the findings related to this study are available from the corresponding authors upon reasonable request.

Human research participants

Policy information about studies involving human research participants and Sex and Gender in Research.

Reporting on sex and gender	n/a
Population characteristics	n/a
Recruitment	n/a
Ethics oversight	n/a

Note that full information on the approval of the study protocol must also be provided in the manuscript.

Field-specific reporting

Please select the one below that is the best fit for your research. If you are not sure, read the appropriate sections before making your selection.

☒ Life sciences ☐ Behavioural & social sciences ☐ Ecological, evolutionary & environmental sciences

For a reference copy of the document with all sections, see nature.com/documents/nr-reporting-summary-flat.pdf

Life sciences study design

All studies must disclose on these points even when the disclosure is negative.

Sample size	No sample size calculation was performed prior to any experiments. Most experiments shown that were aimed at answering new questions relating to the study were performed at least three times independently. Sample sizes were chosen based on technical difficulty and robustness of method, informed by both literature and the authors' experience. Where possible, aggregated data from replicate experiments have been shown in the figures. In other cases, findings from representative experiments have been shown. In the case of 'control' experiments aimed at validating known phenotypes (such as transcription recovery defects after UVC treatment in in NER-deficient cell lines), two replicate experiments were deemed sufficient to confirm these phenotypes.
Data exclusions	In RRS assays, normalised EU intensity values exceeding 200% were considered outliers and excluded from visualisation but not from subsequent calculation of relevant means. These assays aimed at identifying recovery towards 100% after conditions that brought values down to around or below 10%. The outliers above 200% were considered likely to largely arise from microscopy artefacts and were therefore excluded to enable proper visualisation and interpretation of the actual window of biological events. For PxP-MS data analysis, one replicate (#3) out of four was excluded from downstream analysis, due to an overall lower number of identified proteins.
Replication	This study has benefited from the collaboration of two independent research groups with supporting collaborations with several others. Between the two primary groups, the phenotypes representing the key findings (RRS assays and cell viability assays) of the study were independently reproducible between both groups. These approaches were repeated at least 3 times independently between the two main groups, and in some cases cross-validated in additional cell lines (RPE1 and HAP1).
Randomization	No randomization of samples was relevant to the study since all experiments were internally controlled.
Blinding	Blinding was not relevant to the study because as outlined in the Methods section, all quantification and analyses of cell survival and microscopy-based assays were performed using software to apply identical image processing and analysis parameters across samples within each experiment.

Reporting for specific materials, systems and methods

We require information from authors about some types of materials, experimental systems and methods used in many studies. Here, indicate whether each material, system or method listed is relevant to your study. If you are not sure if a list item applies to your research, read the appropriate section before selecting a response.

Materials & experimental systems

n/a	Involved in the study
☐	☒ Antibodies
☐	☒ Eukaryotic cell lines
☒	☐ Palaeontology and archaeology
☒	☐ Animals and other organisms
☒	☐ Clinical data
☒	☐ Dual use research of concern

Methods

n/a	Involved in the study
☒	☐ ChIP-seq
☒	☐ Flow cytometry
☒	☐ MRI-based neuroimaging

Antibodies

Antibodies used

Details of antibodies used can be found in Supplementary Table 7. Rat anti-SPRTN (6F2) is a custom antibody generated at LMU Munich. To request this antibody, please contact Julian Stingele.

Validation

The following antibodies were validated by western blot in knockout cells or with siRNA-mediated depletion: anti-CSB (Abcam ab96089), anti-CSA (Abcam ab137033), anti-SPRTN (Custom, produced at LMU Munich, see Zhao et al (2021) Nucleic Acids Research), anti-RNF4 (R&D Systems AF7964 and Proteintech 17810-1-AP), anti-XPG (Bethyl A301-484A-2).

The following antibodies were not validated by us but behaved as expected (based on available literature) and are commonly used in Western blots: anti RNAPII-CTDpSer5 4H8 (Abcam ab5408), Anti-RNAPII-CTDpSer2 3E10 (Millipore 04-1571), Anti-RNAPII-pCTDpSer2 (Novus BiologicalsNB100-1805), anti-RNAPII-NTD (Cell Signaling 14958), anti-GAPDH 14C10 (Cell Signaling 2118), anti-GFP (Roche 11814460001), anti-ATF3 (Abcam ab207434), anti-Tubulin (Sigma-Aldrich T6074 and T6199).

The following antibodies were used for immunofluorescncе and/or PLA experiments, consistent with prior use in the literature and within the manufacturers' validation statements: anti-GFP (Roche 11814460001), Anti-RNAPII-CTDpSer2 (Novus Biologicals NB100-1805), anti-NPM1 (Abcam ab10530).

The following antibody was validated for use in immunoprecipitation, in agreement with previous literature and the manufacturer's website: anti-RNAPII-CTDpSer2 (Abcam ab5095).

The following antibodies were validated for use in CUT&Tag experiments: anti-RNAPII-CTDpSer5 (Cell Signaling 13523), anti-RNAPII-CTDpSer2 (Cell Signaling 13499).

Eukaryotic cell lines

Policy information about cell lines and Sex and Gender in Research

Cell line source(s)

K-562 cells are human chronic myeloid leukaemia (CML) cells from a female patient, HAP1 cells are derived from KBM-7 cells, which are CML cells from a human male. RPE-1 cells are human female cells derived from the retinal pigment epithelium, MRC5 cells are lung fibroblasts derived from a human male, CS1AN cells are human skin fibroblasts from a female with Cockayne syndrome, and U2OS cells are osteosarcoma cells from a human female. HeLa cells are cervical cancer cells from a human female. Individual cell line sources are as follows:
K562 dCas9-KRAB - Kind gift from Jonathan Weissman
HAP1 WT - Horizon Discovery (Cat no. C631)
HAP1 CSB-/- - Horizon Discovery (Cat no. HZGHC000422c011)
HAP1 XPA-/- - Horizon Discovery (Cat no. HZGHC000433c001)
HAP1 CSB-/- +EV TET3G - This study
HAP1 CSB-/- +CSB-WT TET3G - This study
HAP1 CSB-/- +CSB-K538R TET3G - This study
RPE1-TetOn-Cas9-PuroS-TP53-/- ('WT') - Van der Weegen et al, NCB 2021
RPE1-TetOn-Cas9-PuroS-TP53-/-CSB-/- ('CSB-/-') - Van der Weegen et al, NCB 2021
RPE1-TetOn-Cas9-PuroS-TP53-/-XPC-/- ('XPC-/-') - Van der Weegen et al, NCB 2021
RPE1-TetOn-Cas9-PuroS-TP53-/-XPC-/-XPA-/- ('XPC-/-XPA-/-') - Van den Heuvel et al, PNAS 2023
RPE1-TetOn-Cas9-PuroS-TP53-/-XPC-/-CSB-/- ('XPC-/-CSB-/-') - Van den Heuvel et al, PNAS 2023
RPE1-TetOn-Cas9-PuroS-TP53-/-SPRTN ΔC clone 2 ('SPRTN ΔC#2') - This study
RPE1-TetOn-Cas9-PuroS-TP53-/-SPRTN ΔC clone 4 ('SPRTN ΔC#4') - This study
RPE1-TetOn-Cas9-PuroS-TP53-/-SPRTN ΔC clone 5 ('SPRTN ΔC #5') - This study
RPE1-TetOn-Cas9-PuroS-TP53-/-CSB-/-SPRTN ΔC clone 2 ('SPRTN ΔC#2') - This study
RPE1-TetOn-Cas9-PuroS-TP53-/-CSB-/-SPRTN ΔC clone 10 ('SPRTN ΔC#10') - This study
RPE1-TetOn-Cas9-PuroS-TP53-/-CSB-/-SPRTN ΔC clone 23 ('SPRTN ΔC #23') - This study
RPE1-TetOn-Cas9-PuroS-TP53-/-ELOF1-/- ('ELOF1-/-') - Van der Weegen et al, NCB 2021

RPE1-TetOn-Cas9-PuroS-TP53-/-CSA-/- ('CSA-/-') - Van der Weegen et al, NCB 2021
RPE1-TetOn-Cas9-PuroS-TP53-/-UVSSA-/- ('UVSSA-/-') - Van der Weegen et al, NCB 2021
RPE1-TetOn-Cas9-PuroS-TP53-/-ERCC1-/- ('ERCC1-/-') - Apelt et al, JEM 2020
RPE1-TetOn-Cas9-PuroS-TP53-/-XPG-/- ('XPG-/-') - This study
RPE1-TetOn-Cas9-PuroS-TP53-/-CSB-/- ('CSB-/-+GFP-EV') - This study
RPE1-TetOn-Cas9-PuroS-TP53-/-CSB-/- ('CSB-/-+GFP-CSB-WT') - This study
RPE1-TetOn-Cas9-PuroS-TP53-/-CSB-/- ('CSB-/-+GFP-CSB-K538R') - This study
MRC5 - ATCC (CCL-171)
CS1AN - Kind gift from Alan Lehmann
U2OS - ATCC (HTB-96)
U2OS GFP - This study
U2OS GFP-DNMT1 - This study
HeLa WT - Kind gift from Tomoo Ogi
HeLa CSB-/- - Kind gift from Tomoo Ogi
HeLa RPB1-K1268R - Kind gift from Tomoo Ogi

Authentication

None of the cell lines have been formally authenticated.

Mycoplasma contamination

The cell lines in this study have not all been formally confirmed as mycoplasma-free.

Commonly misidentified lines
(See ICLAC register)

None of the cell lines used in this study are in the ICLAC register.
