# Supplementary figures and images for: Transcription-coupled repair of DNA–protein cross-links depends on CSA and CSB
Source: Nat Cell Biol. 2024 Apr 10;26(5):797–810. doi: 10.1038/s41556-024-01391-1 (PMC11098753; doi:10.1038/s41556-024-01391-1)

Source Data Fig. 1. Unprocessed Western blot images relating to Figure 1

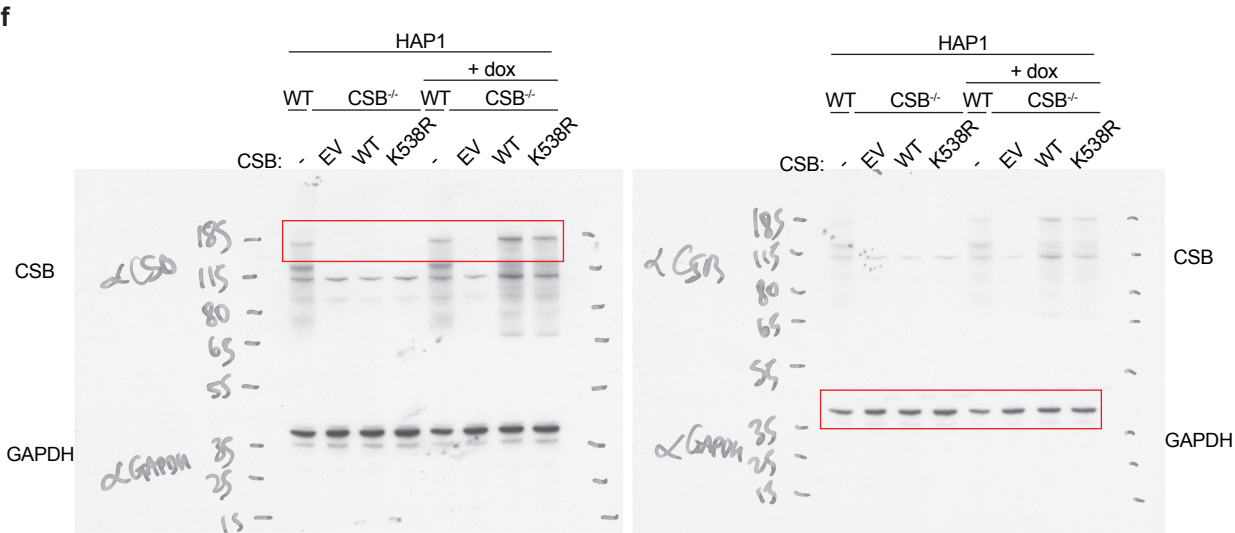

Supplement: Supplementary file 10 — Unprocessed western blots. [file 41556_2024_1391_MOESM10_ESM.pdf]

Source Data Fig. 3. Unprocessed Western blot images relating to Figure 3

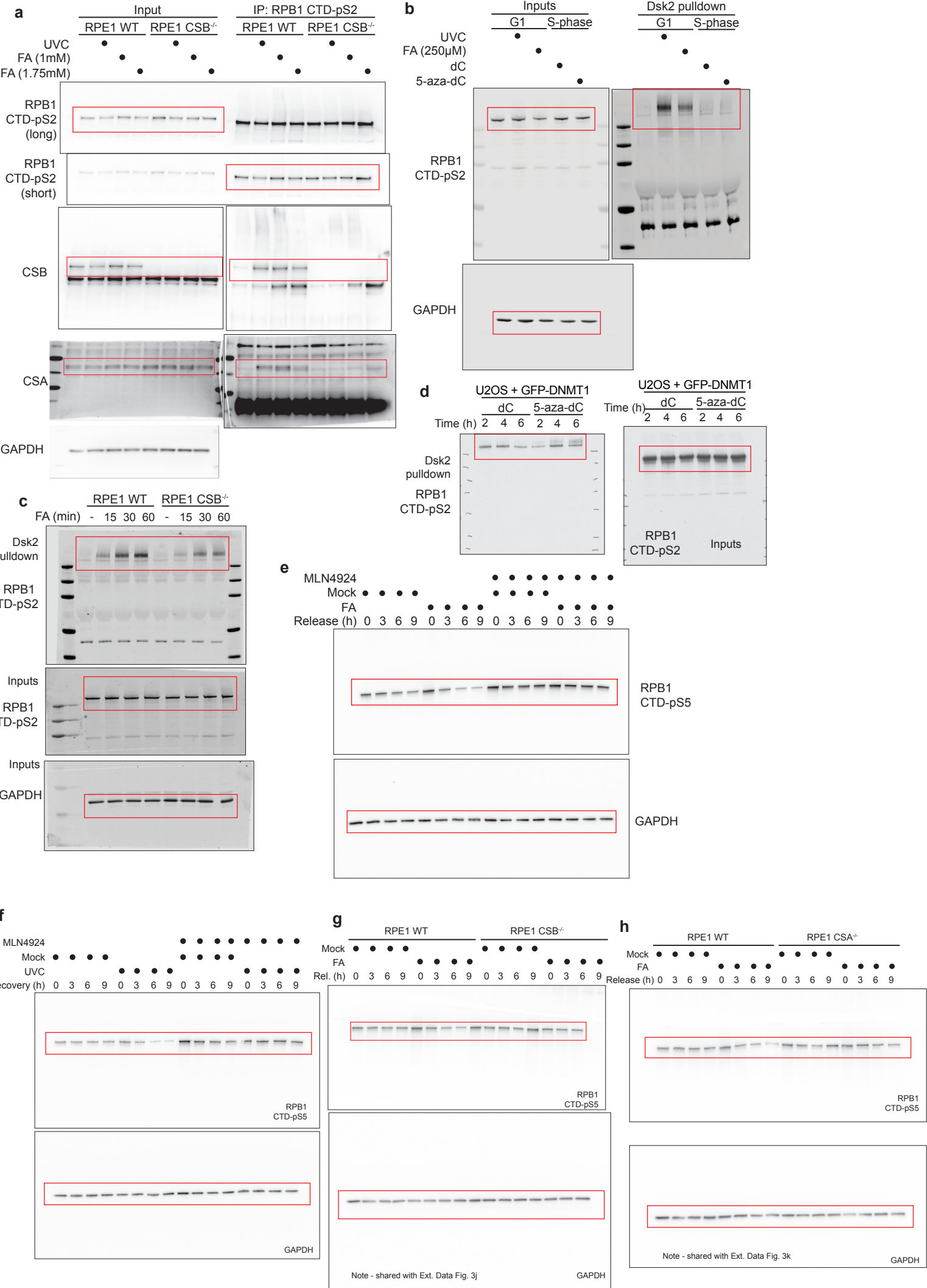

Supplement: Supplementary file 11 — Unprocessed western blots. [file 41556_2024_1391_MOESM11_ESM.pdf]

Source Data Fig. 4. Unprocessed Western blot images relating to Fig. 4

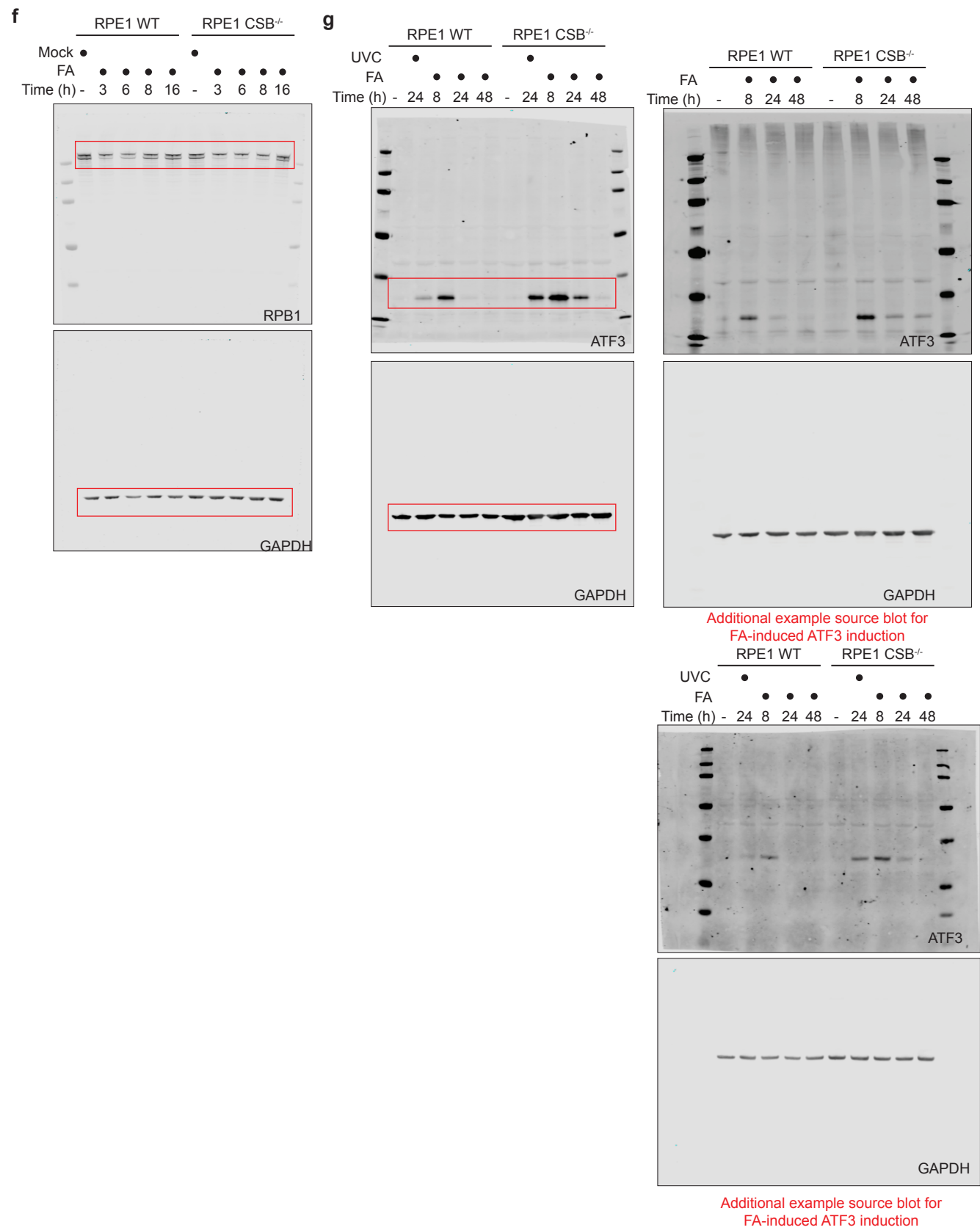

Supplement: Supplementary file 12 — Unprocessed western blots. [file 41556_2024_1391_MOESM12_ESM.pdf]

Source Data Extended Data Fig. 2. Unprocessed Western blot images relating to Extended Data Fig. 2

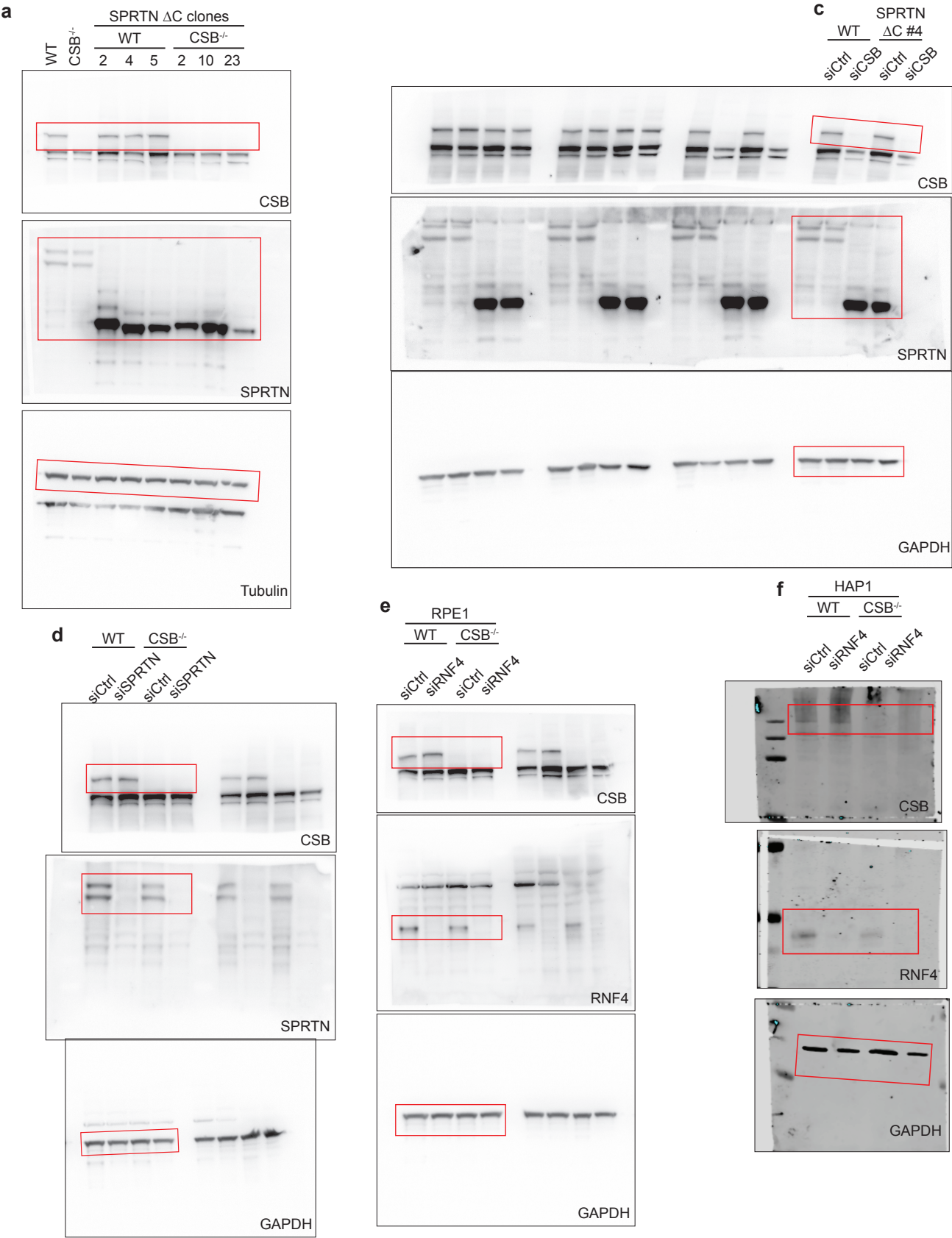

Supplement: Supplementary file 13 — Unprocessed western blots. [file 41556_2024_1391_MOESM13_ESM.pdf]

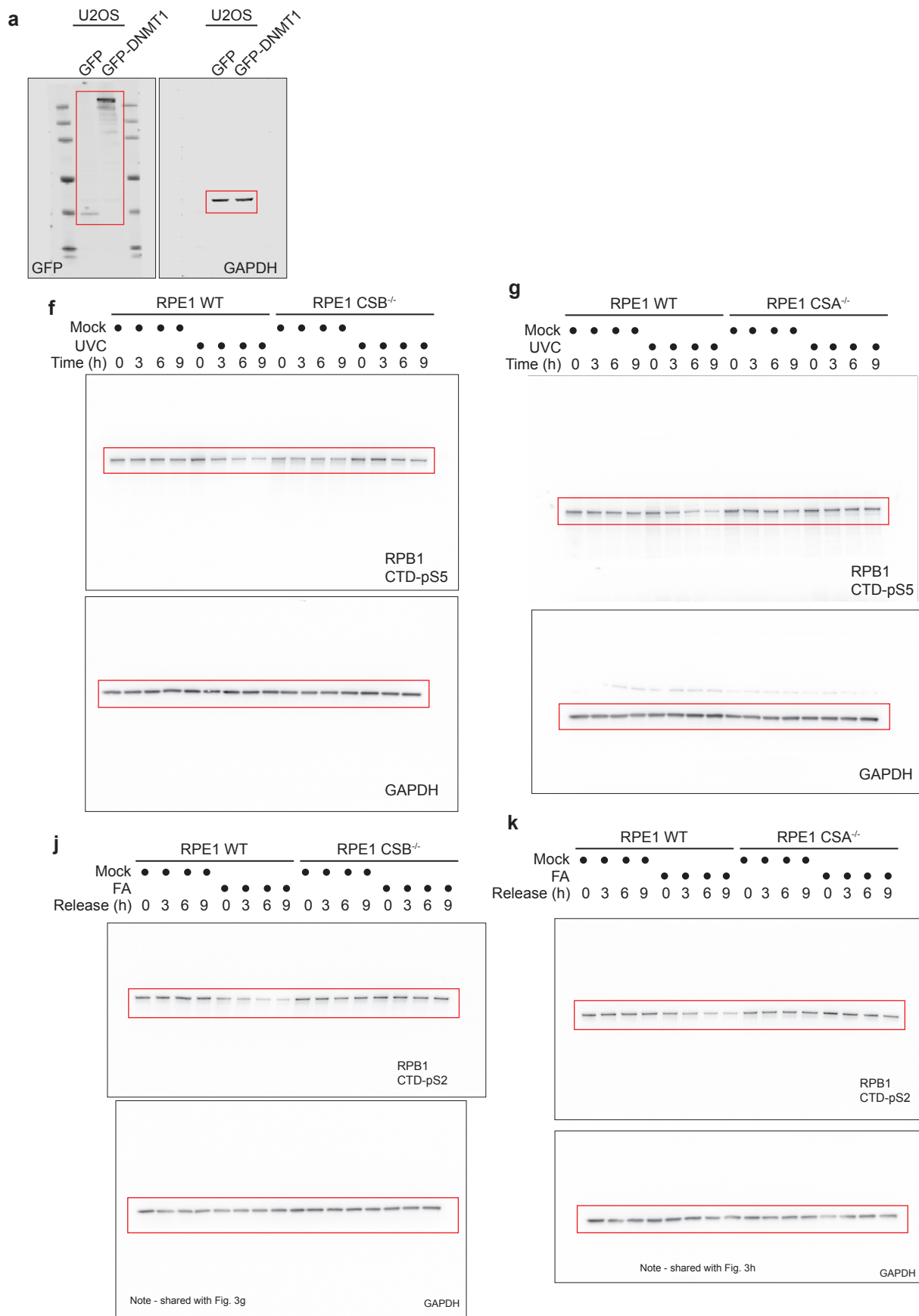

Supplement: Supplementary file 14 — Unprocessed western blots. [file 41556_2024_1391_MOESM14_ESM.pdf]

Source Data Extended Data Fig. 5. Unprocessed Western blot images relating to Extended Data Fig. 5

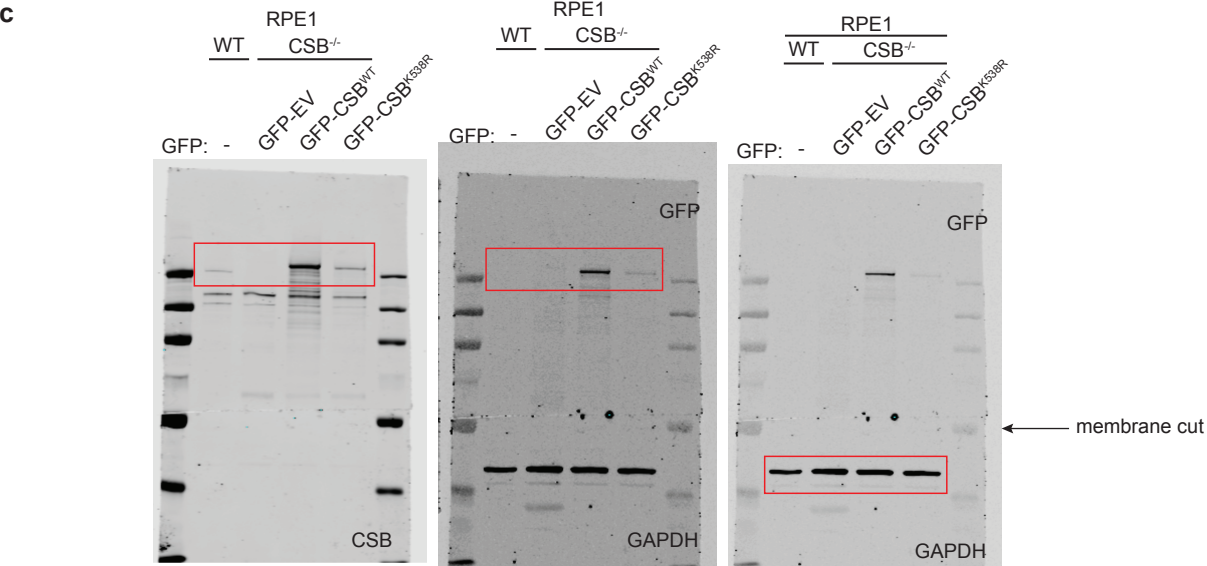

Supplement: Supplementary file 15 — Unprocessed western blots. [file 41556_2024_1391_MOESM15_ESM.pdf]

Source Data Extended Data Fig. 6. Unprocessed Western blot images relating to Extended Data Fig. 6

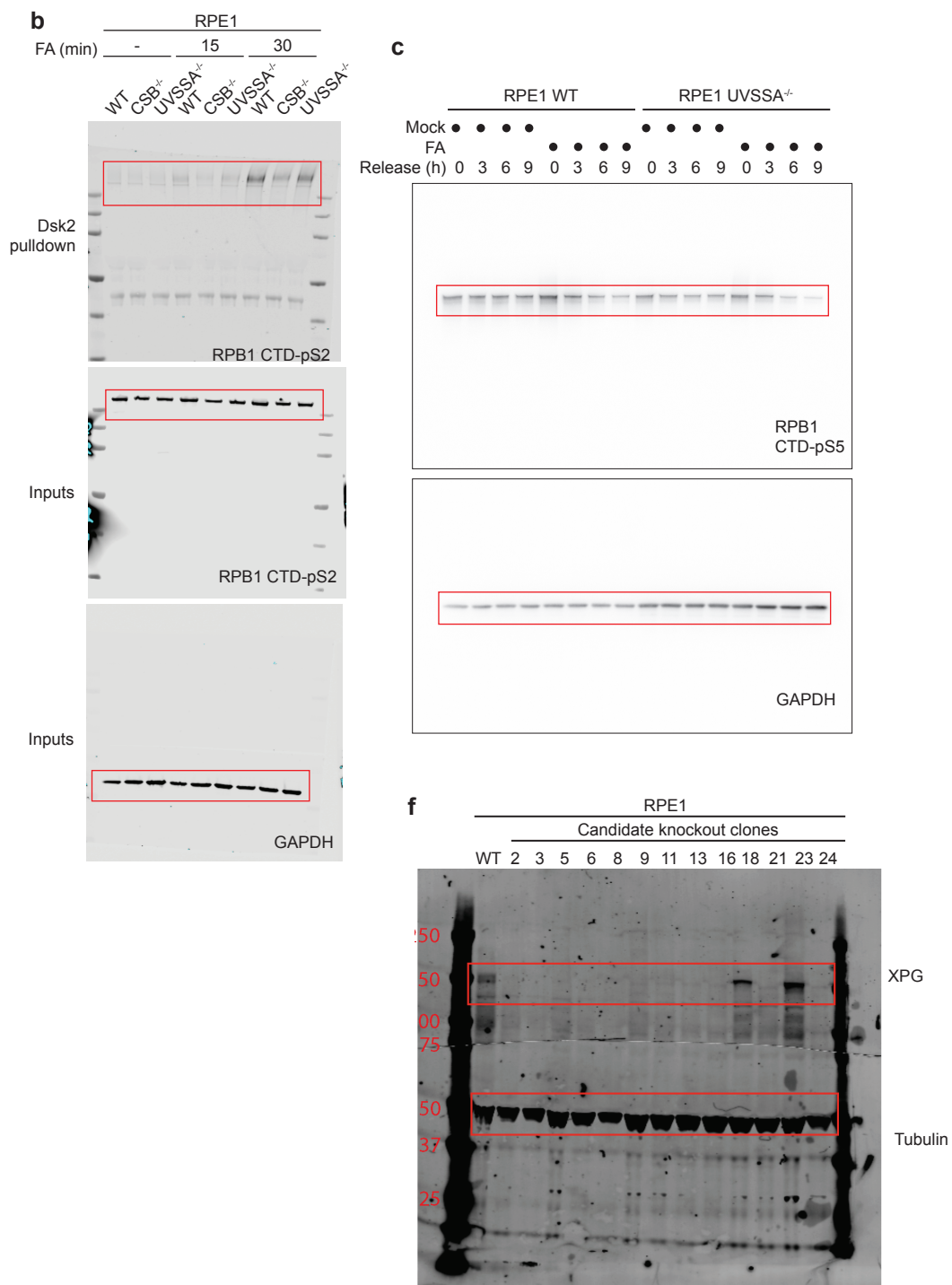

Supplement: Supplementary file 16 — Unprocessed western blots. [file 41556_2024_1391_MOESM16_ESM.pdf]

Source Data Extended Data Fig. 8. Unprocessed Western blot images relating to Extended Data Fig. 8

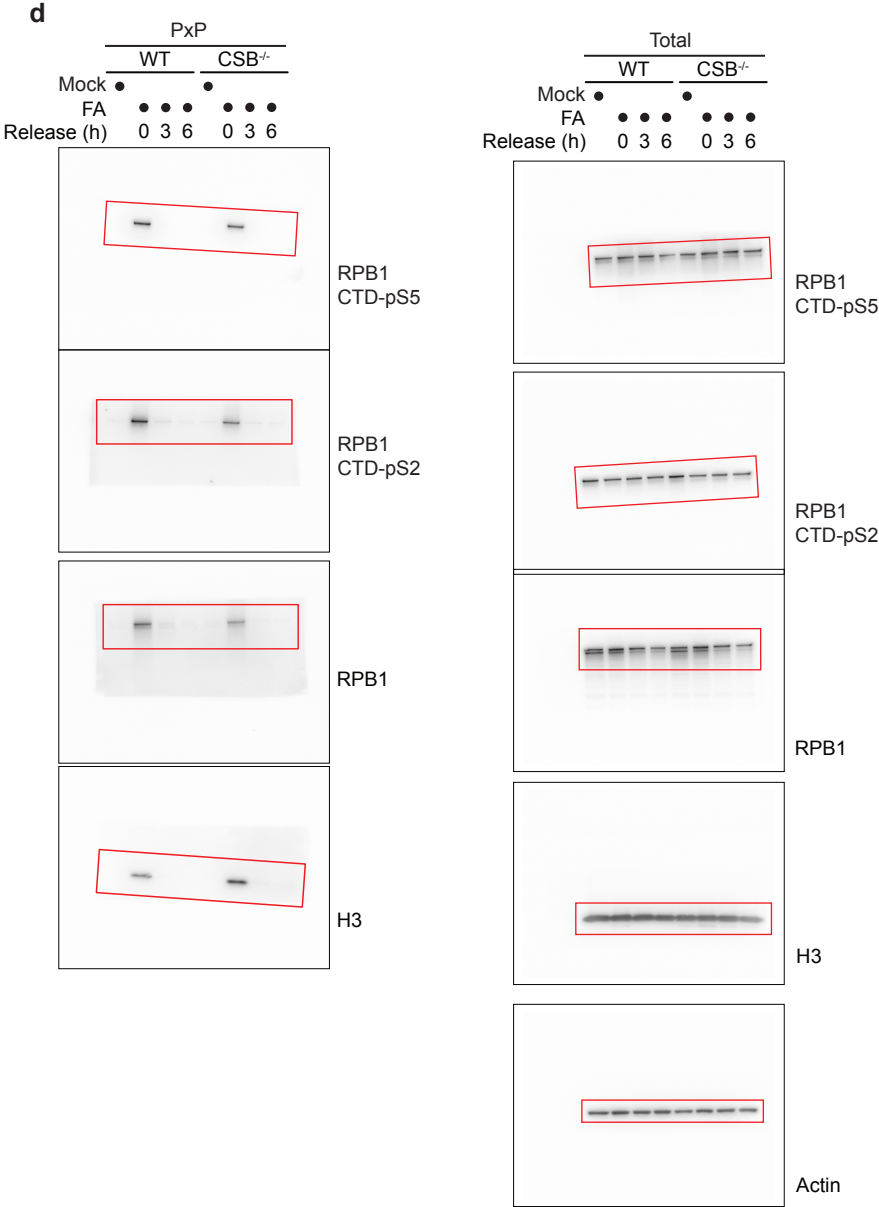

Supplement: Supplementary file 17 — Unprocessed western blots. [file 41556_2024_1391_MOESM17_ESM.pdf]
